# Studying of Membrane Localization of Recombinant Potassium Channels in E.coli

**Published:** 2009-04

**Authors:** O. Nekrasova, A. Tagway, A. Ignatova, A. Feofanov, M. Kirpichnikov

**Affiliations:** 1 Shemyakin-Ovchinnikov Institute of Bioorganic Chemistry, Russian Academy of Sciences, ul. Miklukho-Maklaya 16/10, 117997, Moscow, Russia;; 2 Biological Faculty, Lomonosov Moscow State University, Vorobyevi Gori 1, Moscow, 119992, Russia

## Abstract

The effective expression of recombinant membrane proteins in E.coli depends upon the targeting and insertion of proteins into the cellular membrane, as well as on those proteins adopting the correct spatial structure. A significant technological problem involves the design of approaches for detecting the location of target proteins within a host cell. Using a hybrid potassium channel KcsA-Kv1.3 as a model, we developed a technological scheme which is suitable for the study of membrane localization in E.coli cells of recombinant proteins containing voltage-gated eukaryotic potassium channels as the functional active site. The scheme involves both biochemical and fluorescent methods for detecting target proteins in the cytoplasmic membrane of E.coli, as well as the study of the ligand-binding activity of membrane-embedded proteins.

## 

Studies on trans-membrane proteins and membrane-bound proteins are one of the current trends in biology. Membrane proteins participate in most cellular processes - signal reception and intercellular communications, molecular and ionic transport- and they play a role in the pathogenesis of many diseases and, as such, are the targets for most pharmaceutical preparations [1]. 

Because of the low level of biosynthesis of many membrane proteins in biological tissues, the main source of these proteins for structural-functional studies is from recombinant molecules produced in various systems for heterologous expression [2]. Bacterial cells (in particular, Escherichia coli) represent the most widely used, and most productive, system for the biosynthesis of recombinant membrane proteins [3]. At the same time, the heterologous expression in E. coli of membrane proteins is associated with numerous problems involving the general toxicity of these proteins to the host cells. Besides, recombinant proteins are often produced in aggregated form (with inclusion bodies) necessitating careful preparation to refold such proteins. It would seem more practical to work out an approach for the functional expression of membrane proteins in a bacterial membrane [4]. 

The development of such an approach can be facilitated with the help of simple and effective tests to ensure the correct folding of the target protein within the cellular membrane. These tests, for example, can be based on measuring the functional activity of the protein, or its ability to bind ligands. Furthermore, simple biochemichal assays for determining the location of the target proteins within the cell enable one to more accurately control the insertion of the target protein into the membrane. Such approaches will increase efficiency in the functional expression of target membrane proteins. 

To develop an approach for the controlled functional expression of recombinant membrane proteins in E. coli, we used a hybrid potassium channel KcsA-Kv1.3, which was successfully expressed in bacterial cells [5, 6]. This, and also hybrids KcsA-Kv1.X, which are similar, have been obtained by insertion of the ligand-binding site of eukaryotic Kv1 channels into a homologous site on the bacterial channel KcsA.

Eukaryotic voltage-gated potassium channels like Kv1 are known for their important role in the propagation of nerve impulses, in the regulation of muscle contractions, and in the proliferation of cells [7]. Now, channel Kv1.3 is also being considered as a therapeutic target in the treatment of various autoimmune disorders [8], and the testing of its ligand-binding activity provides the basis for the development of new medications [9].

Production of hybrid proteins KcsA-Kv1.X seemed quite possible due to a high structural and functional homology with potassium channels. These channels are tetramers composed of four α-subunits, each one containing six (voltage-gated eukaryotic channels) or two (bacterial channels) transmembrane helices. In the case of eukaryotic channels, C-terminal helices S5 and S6, which are connected by a loop, form the pore domain, which catalyzes the transport of potassium ions [10]. The bacterial potassium channel, KcsA [11], which has a more simple structure involving an α-subunit, shares a high degree of homology with the pore domains from various bacterial and eukaryotic voltage-gated channels. The most homologous of these is an amino acid sequence comprising a pore loop, which connects transmembrane helices M1 and M2 (Fig. 1a).

**Fig. 1.**
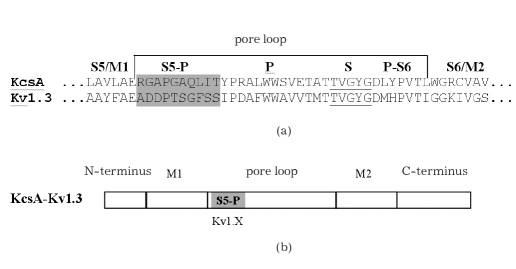
Homology of amino acid sequences between the pore loop of eukaryotic voltage-gated channel Kv1.3 and the bacterial channel KcsA (a) and the schematic representation of the hybrid protein molecule KcsA-Kv1.3 (b). S5/M1 and S6/M2 - transmembrane helices; S5-P and P-S6 -linkers; P - pore helix; S - a sequence for selectivity filter (underlined).

The S5-P linker sequence of eukaryotic Kv1 channels participates in the formation of a ligand-binding site for peptide toxins - the natural blockers of voltage-gated channels [12]. The possibility of replacing the S5-P KcsA linker with the corresponding linker from Kv1.3 resulted in the formation of hybrid protein KcsA-Kv1.3 (Fig. 1b), which represents a receptor with a very high affinity for peptide toxins [5]. This hybrid acquired ligand-binding specificity for toxins, which is inherent to eukaryotic cells as a model system. Channel Kv1.3 is thus a suitable bioengineered protein to be used in the search for new modulators of the activity of the Kv1.3 channel.

KcsA-Kv1.3 was chosen for the present study primarily because of its rather high level of biosynthesis in E.coli (about 2.5 mg/l [5]), which permits the detection of this protein in a cell lysate by means of a denaturing electrophoresis in PAGE. Secondly, the tendency of hybrid proteins to binding labeled ligands can be used for developing alternative methods of membrane protein localization.

The aim of the present work study is to develop a technological approach for achieving the functional expression of recombinant membrane proteins under the control of fluorescent methods. 

## Materials and Methods

### Materials

Reagents were from Merck and Sigma; detergents Mega 9 and N-lauroylsarcozine, sodium salt, were from Amresco; Triton X-100 was from Merck; kits for purification of plasmid DNA, isolation of PCR fragments and extraction of DNA from agarose gel were from Qiagen; restriction endonucleases and DNA-modifying enzymes were from Fermentas. 

### Methods

General protocols for recombinant DNAs were followed in accordance to [13].

The KcsA expression was conducted in E.coli strain BL21(DE3) (Novagen). Overnight culture of E. coli previously transformed with the target plasmid was inoculated in 30 ml of M9 medium enriched with kanamycin (40 mkg/ml) to an initial density of 0.25 at OD_560_. The culture was induced at an OD_560_ of about 1.0 with 50 µM of IPTG, and it was grown with shaking at 37°C for 18 h. The level of hybrid protein expression was analyzed through 13.5% SDS-PAGE [14].

Immunoblotting. Proteins from SDS-PAGE were transferred onto a nitrocellulose BA-85 filter (Schleisher and Schuell) by means of electrophoresis on a Mini-Protean 3 Cell (Bio-Rad) in 25 mM NaHCO_3_, pH 9.2, 10 % methanol for 1.5 h with a current of 0.8 mA per cm at a temperature of 20 °C.

Immunochemical staining was carried out using monoclonal mouse IgG1 antibodies (Penta-His, Qiagen) diluted to 1:1000, and secondary anti-mouse antibodies conjugated with HRP (Novagen) diluted to 1:3000, according to the manufacturer's instructions. TMD (tetramethylbenzydin) was used as a chromogene in a 0.03% solution of hydrogen peroxide. 

Cell fractioning. Probes for fractioning were prepared by pelleting cells from 30 ml of culture (4000 × g, 10 min, 10 °C). Cells were lysed in 4 ml of buffer A (100mM NaCl, 5mM KCl, 50mM Tris pH 8.0) containing 0.5 mg/ml of lysozyme, 1 mM EDTA and 1mM PMSF at 4 °C for 20 min, sounded for 5 min on a Digital Sonifier model 250 (Branson) with 10s pulses at an output of 200 W.

The suspension was centrifuged for 10 min at 10,000 g and a temperature of 5 °C, a pellet was removed (pellet I), and the supernatant was further centrifuged for 1 h at 80,000 g at 5 °C on a TLA 100 (Beckman) producing pellet II. Each pellet was extracted with buffer B (0.1 M Na_3_PO_4_, pH 7.0, 5mM KCl) containing 40 mM Mega 9 (1% of N-lauroylsarcozine or 1% Triton X-100) with gentle shaking for 3 - 4 h, then the suspensions were re-centrifugated, and the final pellets were dissolved in an electrophoresis buffer.

Carbonate wash. Cells were lysed in buffer C (50 mM Tris pH 8, 5 mM EDTA) containing 1mg/ml of lysozyme. The suspension was frozen and thawed 3 consecutive times and then sounded for 1 min with 10 s pulses. Then, an equal volume of 0.2 M Na_2_CO_3_, pH 12, was added to it, and the mixture was incubated on ice for 5 min. Integral membrane proteins were pelleted by centrifugation for 1 h at 80,000 g at 4 °C. The obtained pellet was dissolved in an electrophoresis buffer.

Protein purification by means of metal-affinity chromatography. Protein extracts of pellets I or II were applied onto a 1ml-column of Ni-NTa agarose (Qiagen) equilibrated in buffer B with 40 mM Mega 9. The resin was washed with a solution of 20 mM imidazole, and the target protein was eluted with 0.4 M imidazole in the same buffer. 

Preparation of spheroplasts and binding procedure. Host strain BL21(DE3), carrying pETKcsA-Kv1.3 plasmid, was cultivated, following induction, with IPTG at 37 °C for 18 h in a minimal M9 medium. The cells were harvested by centrifugation (5000 ×g, 10 min, 4 °C), incubated in buffer A (10 mM Tris-HCl, pH 8.0, 0.5 M sucrose, 0.3 mM EDTA) containing lysozyme (20 µg/ml) for 20 min and then stabilized by the addition of MgCl_2_ to a final concentration of 10 mM. 

Obtained in this way, the spheroplast suspension with OD_560_ of about 0.5 was diluted 100-200 times, transferred into the 12-well flexiPERM silicon chamber (Perbio, Aalst, Belgium) attached to a thin (0.17 mm) glass slide and incubated with Rh-AgTx2 at room temperature on a shaker for 1.5 h. The concentration of Rh-AgTx2 (AgTx2 labeled with 5(6)-carboxytetramethylrhodamine-N-succinilmidyl ester) was 10 nM. (Rh-AgTx2 was kindly provided by Yu.V. Korolkova, IBCH RAS). The cells were then analyzed using the inverted laser scanning confocal microscope (LSM510-META, Zeiss, Germany). 

The confocal fluorescent images of spheroplasts stained with Rh-AgTx2 were measured with the C-Apochromat water immersion objective (63×, NA = 1.2, Zeiss) at approximately 0.25 µm lateral and 0.5 µm axial resolution. The fluorescence of Rh-AgTx2 was excited with a He,Ne laser (543.5 nm, 12 µW on the sample), and emission was registered on a 585-nm long pass filter. 

## RESULTS AND DISCUSSION

### Studying the cellular localization of KcsA-Kv1.3 by means of fractioning and detergent extraction 

A gene for the hybrid KcsA-Kv1.3 protein was constructed according to the specifications in [5] and cloned into plasmid pET28a (Novagen). Gene expression was then carried out in E. coli BL21(DE3). The level of expression was determined by analyzing the total protein content with SDS-PAGE, and the presence of the target protein was confirmed by means of immunoblotting with anti-His antibodies to the C-terminal hexahistidine tag. 

It is known that recombinant KcsA and also a hybrid protein KcsA-Kv1.3 accumulate in the cytoplasmic (inner) membrane of E.coli [5, 15, 16]. So, for this reason, isolation of hybrid proteins [5] was carried out from the membrane fraction of cells obtained by high-speed centrifugation of cellular lysate (110 000 g, 45 min). We decided to use this procedure for working out the fractioning procedure aimed at the analysis of the cellular localization of the target membrane proteins - the hybrid potassium channels. 

**Fig. 2.**
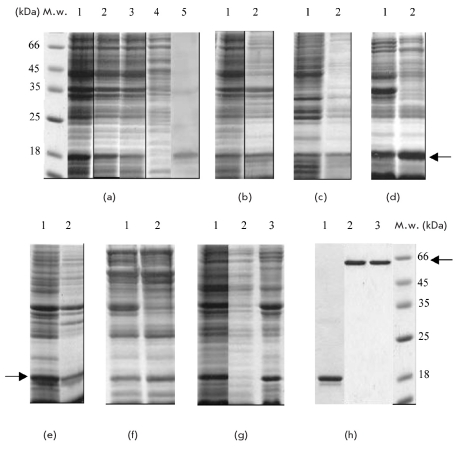
Fractioning of BL21(DE3) cells producing KcsA-Kv1.3 (a). 1 -cellular lysate; 2 - pellet I; 3 - pellet II; 4 - supernatant following high-speed centrifugation; 5 - immunoblotting of cellular lysate with anti-Hisx6 antibodies. Extraction of pellet I with detergent solutions: 40 mM Mega 9 (b), 1% lauroylsarcosine (c); 1% Triton X-100 (d). Extraction of pellet II with detergent solutions: 40 mM Mega 9 (e), 1% Triton X-100 (f). 1 - suspension of pellet in a detergent solution; 2 - extract a Fractioning of cells by the "carbonate wash" method (g). 1 -cellular lysate; 2 - supernatant following high-speed centrifugation; 3 - a pellet. Analysis of KcsA-Kv1.3 tetramer formation (h). 1 - KcsA-Kv1.3 preparation preheated for 10 min at 96 °C; 2 - KcsA-Kv1.3 preparation isolated from the pellet I by extraction with 40 mM Mega 9 preheated for 10 min at 37 °C; 3 - KcsA-Kv1.3 isolated from pellet II prepared under the same conditions. M.w. - molecular weight markers. The position of a target protein (monomer or tetramer) is identified by an arrow.

The procedure of fractioning the cells grown after induction was based on a sounding of cells, pelleting of cellular debris and insoluble components with low-speed centrifugation (10 000 g, for 10 min) (pellet I), and subsequent pelleting of membrane vesicules by high-speed centrifugation (pellet II). As shown in Fig. 2a, hybrid protein KcsA-Kv1.3 is contained within an insoluble cell fraction, and a considerable amount of it is detected in pellet II (Fig. 2a, lane 3), suggesting that KcsA-Kv1.3 is inserted into the cellular membrane. Noteworthy, recombinant KcsA-Kv1.3 protein is found in the pellet I (Fig. 2a, lane 2). This low-speed pellet is usually considered as a fraction of inclusion bodies - insoluble protein aggregates of the target protein formed by the nascent chains of recombinant proteins in denatured form [17]. In the case of soluble protein expression, the isolation of active proteins from inclusion bodies by means of a re-naturing procedure is carried out. In the case of membrane protein biosynthesis, the composition of pellet I, as well as the mode of its formation, might be different. So, in work [18] it is shown that the distribution of proteins within the cytoplasmic membrane of E.coli is uneven: with the help of differential centrifugation, "light" membrane fractions without protein, "heavy" membrane fractions overloaded with protein, and intermediate fractions were identified. Taking into account that KcsA-Kv1.3 protein is overexpressed in BL21(DE3) cells, it is conceivable that pellet I contains membrane vesicules saturated with the target protein molecules and, thus, has a higher density than pellet II. This mechanism of pellet I formation is mentioned in review [2]. It cannot be excluded that pellet I contains denatured molecules of the target protein. 

Pellets I and II were studied by means of detergent extraction. For this, nonionic detergent Mega 9 was used, which is usually the detergent of choice for the extraction of KcsA and KcsA hybrid proteins from the membrane fraction [19]. As shown in Figs. 2b and 2c, incubation of each pellet with Mega 9 resulted in the dissolution of the target protein. Then, pellets were subjected to extraction with the mild ionic detergent lauroylsarcosine. Solutions of 1 - 2% lauroylsarcosine are usually used for the selective dissolution of E. coli cytoplasmic membrane [20, 21] and, in some cases, for dissolution of the low-speed pellet to recover a target membrane protein [22, 23]. The target protein was quantitavely extracted from pellets I and II using lauroylsarcosine (results of the dissolution of pellet I are shown in Fig. 2c). Pellets I and II were totally dissolved in 1% solution of Triton X-100 according to [24] (Figs. 2d and 2f). Triton X-100 (in 0.5 - 1 % solutions) is frequently used for dissolving the cellular membrane fraction, as well as for washing the inclusion bodies of any contaminating proteins [17].

To confirm that the target protein was indeed localized in the cellular membrane, we determined the total membrane protein fraction using the method of "carbonate wash" [25]. This method is based on the selective solubility of both cytoplasmic protein aggregates and membrane-bound proteins in an alkaline solution at pH=12, and the subsequent pelleting of integral membrane proteins by high-speed centrifugation. As shown in Fig. 2g, the whole amount of the hybrid KcsA-Kv1.3 is found in the pellet fraction. 

Next, we purified the KcsA-Kv1.3 from the protein extracts on a column of Ni-NTA Sepharose, and then the isolated target protein was analyzed in SDS-PAGE using two protocols - one with preliminary heating at 96 °C for 10 min in a buffer containing 1% SDS, and the other without heating. This test, which is used for determining the native tetramer formation of KcsA and its mutations, is based on the unique thermal stability of the KcsA tetramer in solutions of different detergents [16]. For example, in the 0.1% SDS solution the melting temperature of KcsA is 68 °C. It is clear from Fig. 2h that hybrid protein KcsA-Kv1.3, which is recovered either from pellet I or from pellet II, forms a tetramer. Taking into consideration that the presence of a lipid environment is one of the prerequisites for KcsA tetramer formation [26], and on the basis of the data showing a high degree of solubility of KcsA-Kv1.3 from both pellets in detergents (Mega 9, lauroylsarcosine and Tritone X-100), one can assume that KcsA-Kv1.3 in pellet I is properly folded and integrated into membrane vesicles. Additional confirmation of the presence of a membranous environment in both pellets comes from the location of the whole amount of KcsA-Kv1.3 within the insoluble fraction following the "carbonate wash." The obtained results allow one to correctly evaluate the data from the fractioning of E. coli cells producing membrane proteins, indicating that the location of a target protein within a low-speed pellet (pellet I) does not exclude the assumption, by this protein, of native fold in a membranous or membrane-like surrounding. 

### Detecting the membrane localization of KcsA-Kv1.3 by means of fluorescent microscopy

The presence of a ligand-binding site in the hybrid KcsA-Kv1.3 molecule creates an opportunity to detect the insertion and folding of this recombinant protein into the cytoplasmic membrane of E. coli. For this, we used a procedure for binding KcsA-Kv1.3 to a fluorescently labeled peptide toxin - agitoxin2, on the entire surface of E. coli cells [26]. Agitoxin (AgTx2), a 38-a.a. peptide from the venom of a scorpion, Leiurus quinquestriatus, is an effective blocker of the Kv1.3 channel [27]. Binding to the channel from the outside of the pore, it inhibits the transport of potassium ions through the channel. Agitoxin2 does not exhibit affinity for KcsA, but it effectively interacts with the purified KcsA-Kv1.3 (IC_50_=6.4 nM) [5]. Furthermore, AgTx2 is sensitive to the structural integrity of KcsA, and it is usually used for probing the quaternary (tetramer) structure of this channel [28]. 

**Fig. 3.**
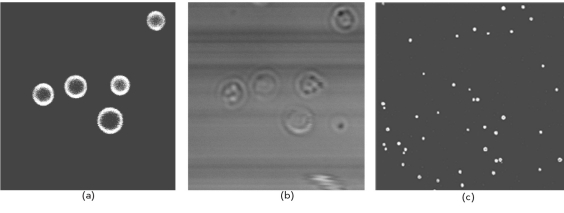
Binding of Rh-AgTx2 to the membrane of spheroplasts. (a, c) Typical confocal fluorescent images of Rh-AgTx2 bound to E.coli BL21(DE3) producing KcsA-Kv1.3. (b) Transparent light image of spheroplasts. Length of the bar corresponds to 5 µm.

For fluorescent detection of binding agitoxin to KcsA-Kv1.3, BL21(DE3) cells with KcsA-Kv1.3 were first converted into spheroplasts by lyzosyme treatment in order to disrupt the cell wall and thus allow a labeled toxin to reach the cytoplasmic membrane. Then, spheroplasts were incubated with agitoxin that had been labeled with a rhodamine dye and subjected to analysis with a scanning confocal microscope. Figure 3 shows that a fluorescent signal is detected at the surface of the cellular bacterial membrane. Control BL21(DE3) lacking a plasmid and BL21(DE3) with the KcsA protein did not demonstrate any binding. These results suggest that hybrid KcsA-Kv1.3 is located in the inner membrane in a functional active form and is able to specifically bind a ligand; in this case, a peptide toxin. 

Our experiments show that the set of methods used (biochemical and fluorescent methods of analysis) allow one to adequately estimate the location of recombinant proteins - hybrid potassium channels, within the cytoplasmic membrane of E. coli. 

## CONCLUSIONS

1. Using the KcsA-Kv1.3 hybrid protein as a model, we developed an approach for the study of the membrane localization, within E.coli cells, of recombinant channels containing functionally active sites composed of eukaryotic, voltage-gated potassium channels. The technical scheme is based on commonly used biochemical techniques, as well as on fluorescent methods of analysis. It includes: - fractioning of E.coli cells, producing recombinant membrane proteins by means of the differential centrifugation of the cellular lysate; - analysis of the pellet fractions with the help of extraction using different detergents; - determination of membrane proteins within the cellular lysate through a "carbonate wash" method; - determination of KcsA-Kv1.3 tetramer formation by means of SDS-PAGE electrophoresis; - detection of the insertion and proper folding of a target protein within the cellular membrane by means of fluorescent methods of analysis using whole E.coli cells. 

2. The present study demonstrates that a low-speed pellet, produced during cell fractioning, contains the target protein, which forms a proper tetramer structure in a membranous surrounding. 
